# “*How can you advocate for something that is nonexistent?*” *(CM16-17)* Power of community in a pandemic and the evolution of community-led response within a COVID-19 CICT and testing context

**DOI:** 10.3389/fpubh.2022.901230

**Published:** 2022-09-21

**Authors:** Sarah J. Hoffman, Yesenia Garcia, Julieta Altamirano-Crosby, Sarait M. Ortega, Kimberly Yu, Seja M. Abudiab, Diego de Acosta, Windy M. Fredkove, Sayyeda Karim, Erin Mann, Christine M. Thomas, Katherine Yun, Elizabeth E. Dawson-Hahn

**Affiliations:** ^1^Population Health and Systems Cooperative, School of Nursing, University of Minnesota, Minneapolis, MN, United States; ^2^University of Washington, Seatle, WA, United States; ^3^WAGRO Foundation, Lynnwood, WA, United States; ^4^Centro Binacional para el Desarrollo Indígena Oaxaqueño, Frenso, CA, United States; ^5^National Resource Center for Refugees, Immigrants, and Migrants, Minneapolis, MN, United States; ^6^Department of Pediatrics, University of Washington, Seatle, WA, United States; ^7^Center for Global Health and Social Responsibility, University of Minnesota, Minneapolis, MN, United States; ^8^Division of Infectious Diseases and International Medicine, Department of Medicine, University of Minnesota, Minneapolis, MN, United States; ^9^Division of General Pediatrics, Children's Hospital of Philadelphia, Philadelphia, PA, United States

**Keywords:** refugee, immigrant, migrant, COVID-19, community, public health

## Abstract

Formal and informal bilingual/bicultural organizations and networks form the backbone of support for refugee, immigrant, and migrant (RIM) communities in the United States. They are pivotal in mitigating barriers and inequities in social and structural determinants of health. These organizations and networks are situated within the communities they serve, and often are established and run by members of a community, to serve the community. In the United States, the COVID-19 pandemic surfaced and widened existing health inequities for some racial and ethnic communities. Our primary objectives were to: (1) describe the processes that underpinned the pivotal role of immigrant-serving community structures in developing and implementing culturally sustaining programming in the context of pandemic response, and (2) amplify the voices of community experts, as they shared experiences and perspectives around these humanistic and community-centered approaches. We applied a community case study approach to a national sample of RIM-serving community structures representing broad country/region-of-origin, cultural, and linguistic identities. Community engagement strategies utilized in the project period included engaging community partners to identify and facilitate connections, and consult on analysis and dissemination. The project team conducted 20 in-depth, semi-structured interviews with a purposive sample of community experts/community organizations. Sampling strategy was further informed by immigrant identity (i.e., characterization of status) and geography (i.e., United States Department of Health & Human Services, Office of Intergovernmental and External Affairs Regions). Through thematic analysis, results identified key contextual, process-, and impact-oriented themes inherent to community-led COVID-19 responses, that were situated within and around the public and health system response to the pandemic. As public health and health systems scrambled to address acute and unprecedented barriers to access, distribution of COVID-19-related health resources and services, and disparate health outcomes, community structures diligently and intentionally reimagined and reconceptualized their response to COVID-19, frequently in the setting of scarce resources. The grassroots response evolved as a counter-narrative to top–down equity processes, historically defined by systems and applied to the community.

## Introduction

This qualitative community case study is focused on the pivotal role of community experts and immigrant-serving community-based organizations in innovating and implementing culturally responsive programming in the context of a public health COVID-19 pandemic response. We leverage the platform of the Centers for Disease Control and Prevention (CDC) funded National Resource Center for Refugee Immigrant and Migrants (NRC-RIM) project to amplify the voices of individuals and organizations who created a roadmap for community-led pandemic response.

Central to our inquiry is the disproportionate impacts of the COVID-19 pandemic on migrant, immigrant, and refugee communities. In the United States, immigration status is an important social determinant of health (1). The COVID-19 pandemic has surfaced, or made visible, and perpetuated health inequities experienced by immigrant communities (2, 3). This is particularly the case in the setting of systemic inequity and the compounding effects of chronic stress and trauma (4). Immigrant populations are underrepresented in disaggregated results of studies examining the impact of the COVID-19 pandemic on priority populations (5–7). In a state-level analysis, Black and Latinx communities experienced statistically significant increases in COVID-19 case rates in the first 5 months of the pandemic, where factors such as “foreign-born noncitizen status,” employment type, and key structural inequities explained the disproportionate impacts observed (8). A health system analysis reported that COVID-19 test positivity rates among non-English language speakers were more than four times the test positivity observed among English language speakers in the sample (9). Underrepresented communities, in particular African American and Latinx communities in the United States, have endured the greatest burden of the pandemic (10). Explanations for the disproportionate impact of the pandemic on communities of color and immigrant communities are expansive and complicated, emphasizing multifactorial and intersectional origins (11).

Partnering with immigrant communities to understand their perspectives and leadership is critical to addressing the health inequities that COVID-19 has caused. This case study is intended to highlight and amplify the perspectives of a diverse sample of community experts based on immigrant communities. Our primary objective was to describe processes that underpinned the pivotal role of immigrant-serving community structures in developing and implementing culturally responsive programming in the context of pandemic response.

## Methods

The parent project is a qualitative initiative situated within the National Resource Center for Refugees, Immigrants and Migrants (NRC-RIM) project based at the University of Minnesota. Key objectives of the parent project were to: (1) explore the perspectives of public health and health system practitioners and community experts on perceived and/or experienced facilitators and barriers of the COVID-19 response in immigrant communities; and (2) inform the development of best and promising practices for case investigations and contact tracing within immigrant communities. In this report, we focus on the subset of the data collected through interviews with refugee, immigrant, and migrant- (subsequently referenced as “immigrant” in this report) serving community experts and key representatives from community organizations (subsequently referenced as “CE/CO” or “interviewee” in this report). We applied a community case study approach to a national sample of immigrant-serving community structures representing broad country/region-of-origin, cultural, and linguistic identities.

### Community consultation

Two community-based consultants were consulted to guide the interpretation of data, analytic decisions, manuscript development, and dissemination. Both identified as members of immigrant communities and held leadership positions in established and active immigrant-serving organizations.

### Ethical considerations

The quality improvement initiative protocol was reviewed and determined not human subjects research by the University of Minnesota and exempt by the University of Washington Institutional Review Boards.

### Interview guide

A semi-structured interview guide was developed by members of the qualitative team and key project stakeholders. The guide was used across data sets in the parent study and was iteratively adapted in the community to reflect ways that CE/CO was engaging with Case Investigation and Contact Tracing (CICT), or the tracking of infection source and spread within networks, and vaccination.

### Sample

The target sample was 20 community experts and/or representatives of immigrant-serving community-based organizations. Through a mix of self-referral and targeted purposive sampling via the networks of the study team (including the NRC-RIM Community Leadership Board), 40 stakeholders representing diverse geographies and backgrounds were invited to participate. Among those contacted for interviews, 19 declined, did not respond, or it was mutually determined that the contact did not meet inclusion criteria. A single organization provided written resources to the team. We framed our sampling strategy to prioritize communities who, to the best of our knowledge, were among the most heavily affected by the pandemic. Potential interviewees were screened and included if they: (1) identified as a member of a refugee, immigrant, or migrant community; and (2) were engaged in the COVID-19 response with immigrant communities. Representation across the 10 US Department of Health & Human Services (HHS), Office of Intergovernmental and External Affairs Regions was prioritized. We categorized organizations as “refugee, immigrant, migrant-specific” if the organization as a whole or the community expert representing the organization self-reported a specific focus on RIM communities. Interviewees received a $40 gift card upon the completion of the interview.

### Procedures

Interviews with CE/COs were conducted from December 2020 to April 2021 via the Zoom platform with members of the qualitative team. Following the completion of a demographic survey, interviewers reviewed the project objectives and procedures with the interviewees and obtained permission to audio record. Semi-structured interviews lasted 60 min and were professionally transcribed. Transcripts were uploaded into a secure file-sharing platform hosted by the University of Minnesota. The interviewer then completed a rapid interview summary based on the transcript for review and discussion by the full qualitative team. All interviews were conducted in the English language. In one case, a group interview included two interviewees; otherwise, interviews were conducted with a single interviewee.

### Analysis

Demographic data were collected and stored using University of Washington REDCap electronic data capture tools (12, 13). Transcripts were uploaded and coded using Dedoose software (14). Initially, a two-member coding team independently double coded five transcripts. Of those five, two were reconciled line by line with a focus on code development. Subsequently, three transcripts were reconciled holistically with a focus on codebook refinement. Deductive codes were established *a priori* in the initial waves of data collection with health system providers and public health officials. Subsequently, inductive codes unique to the CE/CO data set were identified through open coding. Upon the completion of the initial transcripts and preliminary codebook, a single study team member coded the remaining 14 transcripts over a 25-week period (January 2021–July 2021). One transcript was deemed not relevant to the interview set and was transcribed but not coded. Members of the coding team met weekly to review and reconcile the codebook. The full qualitative team met weekly with the coding team and supported key decision points in codebook development.

Thematic analysis (15) functioned as the analytic foundation. Two members of the study team led the analysis. Initially, analysts re-read all interview transcripts. Independently, the analysts organized parent and child codes into themes with a focus on prevalence and keyness to guide decision-making. Analysts met weekly to reconcile code organization and theme identification. Themes were then preliminarily defined and an initial conceptual diagram was drafted to visualize the relationships between themes. Excerpts within each theme were then re-reviewed to establish alignment and consistency within the themes. A multi-layered analytic and methodologic memoing strategy was a prominent analytic tool. Theme names and definitions and the conceptual diagram were finalized. Key excerpts representing each theme were identified and extracted, co-occurring codes were examined for analytic relevance. References in excerpts to specific countries and ethnic groups were deidentified. This decision was made in consultation with community-based manuscript consultants as the study team weighed the balance between acknowledging and preserving unique cultural experiences in the COVID-19 pandemic with privacy and possible potentiation of stigma and harm.

## Findings

Table 1 describes the demographic characteristics of the interviewees. Interviews geographically represented all HHS regions except region 7, there was no representation in this interview set from Iowa, Kansas, Missouri, or Nebraska. A wide range of ethnoracial groups was represented and populations of focus included refugee, immigrant, and migrant communities. The roles interviewees held within their organizations or coalitions were diverse.

**Table 1:** Demographic characteristics of community expert/community organization (CE/CO) participants.

**Characteristics**	**CE/CO numbers**
Total number of interviewees*	22
Location, by HHS region	
1 or 2	3
3 or 4	6
5 or 6	1
7 or 8	1
9 or 10	8
Organizational level	
Local (City/County)	16
State	1
Regional	0
Southern border/state community	2
Organizational type	
Non profit	11
Community based organization	2
Community health/advocacy organization	4
Informal^#^	2
Refugee immigrant migrant (RIM)-specific organization**	19
Populations served***	
Refugees	9
Migrant workers	8
Other immigrants	7
Interview completed after first COVID vaccine Emergcncy Use Authorization (EUA)****	10

*Many organizations requested group interviews with 2 or more staff members.

**We categorized organizations as “refugee, immigrant, migrant-specific” if the organization as a whole or the operational unit within the organization (e.g., a state refugee health program within a Department of Public Health) focuses specifically on RIM communities.

***Many organizations work with more than one population.

****December 11, 2020.

^#^Informal organization type characterized informants that did not report a formal nonprofit designation and/or self-identified as being not affiliated with an existing entity.

**Figure 1:**
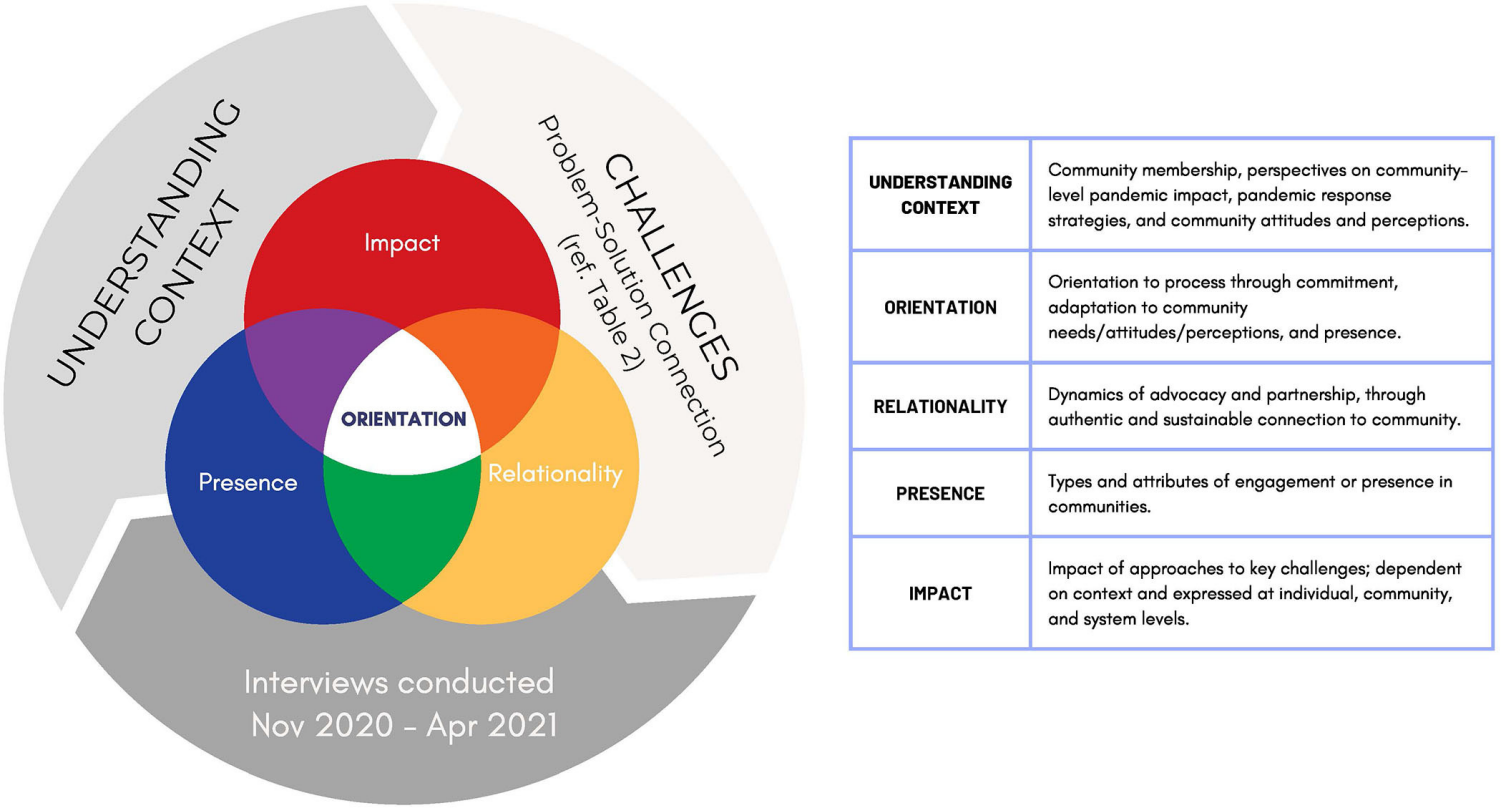
A visual representation of iterative cycle relationships between themes.

Findings are organized by the five themes: understanding context, orientation, relationality, presence, and impact (Figure 1). Themes represent prominent characteristics of a culturally responsive and impactful, community-led pandemic response identified by CE/COs.

### Understanding context

CE/COs provided rich and meaningful descriptions of community membership, perspectives on community-level pandemic impact, COVID-19 pandemic response, and community attitudes and perceptions. In essence, CE/COs described how they understood community context and thus explained the framework through which they approached and supported the community.

#### Community-level pandemic impact

CE/COs described existing systemic inequities that were heightened in the pandemic.

*…most of the time we really are invisible to the systems. Like when we're identified as you're either* [deidentified] *or you're* [deidentified]*, but the whole identity of our communities is erased, as when they label us as those two major groups, not necessarily capturing the diversity within the community, that we're a multi-ethnic and multi-lingual nation. (CM19-21)*

Isolation and the economic impact of the pandemic on immigrant communities were prominent in statements.

*…People who are non-white…it has affected them more than it has affected people who are from a white background. For my community specifically, the loss of jobs. A lot of people have lost their jobs and it really had a ripple effect because they lost their jobs, they were behind on rent, didn't have enough money to buy food, so they relied a lot on food pantries or churches or mosques that offered free food, or the schools that delivered or that offered free lunches for the kids because of home schooling….We're a very communal people. We like to connect, hang out and eat together, pray together, go to naming ceremonies when somebody has a child or when somebody passes away or when somebody is getting married. Those social events were gatherings that had some sort of mental effect, right, creating stress and depression, because you're not connecting with your people like that. You have to be in quarantine. (CM08)*

Underlying these statements was a dual role many CE/COs held as individual members of the community experiencing the challenges of the pandemic and essential community support.

#### COVID-19 pandemic response

CE/COs outlined three iterative phases of community capacity building for pandemic response: (1) functional, (2) operational, and (3) structural. The *functional* phase acknowledged a realization of/preparation for/movement toward activities or positions that were not yet actualized due to insufficient resources. At the point where resources were available, the *operational phase* was how those functional adaptations were put into practice/operationalized in a more systematic and supported way. Operationalizing also moved the practices/programs along the continuum of being available to the community. *The structural* phase represented the framework of response that became embedded within the organization structure and/or layered in a web of response strategies unique to the community.

##### Functional phase

The functional phase described the adaptation that was necessary for CE/COs to be responsive to community needs, particularly as the public health system became overwhelmed. Interviewees described the effectiveness of the CE/CO role as a conduit of information, resources, and connection.

*We basically created another organization within the organization, just to respond to the COVID pandemic. We started with popup food pantries and now we actually are a formal food pantry, one of the largest in the city serving 11,000 people per week. That is with pickup, drive through and home delivery food. Then we also have promotores de salud, health promoters disseminating PPE and testing education and now vaccination education. We also supported with unemployment application assistance, so triaging and making sure that people were applying for unemployment and supporting that process…(CM23)*

Interviews reflected the shifting context of the pandemic. Early on, when testing, treatment, and a fundamental understanding of the virus itself were significantly deficient, fear was prevalent among community members.

*So, I feel that it's been in different stages. In March, when we, as employees, were sent home, we stopped everything that we were doing and started focusing on first learning what COVID was. Because, as everybody else we had no idea. A lot of people were scared, and you can imagine that extra layer of fear in our community, because they're only fearful of getting in trouble here or driving and the police catching me without a driver's license. All that, to then all these shutdowns that happened and people having to go to work and all these things that they were saying, “If we catch you driving after this time…” So, it really affected a lot of people not being able to work and having to stay home, not making money to feed their families or pay bills. So, right after we learned* [what] *COVID was, it wasn't even the fear of getting infected, it was more of—how do I pay my bills? How do I see my family?—that was there, right? So, after that fear kinda, it was like, “Well, this is just something else we've got to work with.” (CM01)*

This statement highlighted the depth of understanding CE/COs had about the concerns that were circulating in communities. The interviewee referenced privilege in the ability to choose to work remotely; whereas that choice may not have been accessible to individuals choosing to work due to personal or economic circumstances

##### Operational phase

The operational phase described how available services and resources were put into action and/or made accessible to the community, e.g., testing, CICT, linguistic services, and education outreach. Where *function* was to understand what the community needed, and when and how to meet that need, *operation* was to set resources in motion. Interviewees described ways that the *functional phase* often guided or preceded the *operational phase*, because there were delays in capacity and resources. The availability of linguistic resources and services underpinned this phase. Interviewees reflected on how language access drove widening health inequities.

*We continued to do education and outreach and we developed materials in Indigenous languages. There's often the belief that you can just write the language, which is true, but more for the academic kind of world and for people that have access to that, but most of our community doesn't know how to read and write in* [deidentified]. *So we worked on a lot of audios, with different organizations, with the state, among ourselves to get the message across and have our staff either send it through our community or have those one-on-one conversations with them as well. That's some of the outreach and education we have done. (CM19-CM21)*

CE/COs described a process-oriented approach to developing effective health communication that was essential for key elements of pandemic response (e.g., CICT, testing, outreach, and concurrent service provision) to be understood, initiated, and sustained.

*You can say in English, “you need to get tested, because you can save lives.” That is core and English-speaking folks that have access to information can be like, yeah, it saves lives. But for working-class Indigenous folks, it's like, I don't have health insurance. Is this going to be free? The message needs to be about access and about it is free. You can do that, but at the same time, for it to be adequate, doing a testing site at 9 AM, it's not going to work for people that now are considered essential and have to work. (CM19-21)*

Interviewees emphasized that language, not just words but also timing, meaning, and delivery, informed effective health communication.

##### Structural phase

The structural phase of pandemic response was realized when an approach became embedded within the organizational structure and/or layered in a web of response strategies unique to the community. A CE/CO described the evolution of the response and ultimately the layering of services and outreach that were part of service provision.

*At first two, three people that went out in the fields, getting up at 7 in the morning to take PPE, to make sure that people got the information they need. It was at first two staff members. Then they noticed everyone was going to be fine. “We can go and be OK,” so then more of our staff kept joining. In* [deidentified] *we were able to go to the fields and visit the workers to ensure that they got not just the PPE, but they had the information they need, the more up-to-date information on COVID development… Then from there we worked with the county health department to get a contract to do not only education and outreach but contact tracing, that we were offering that language access support at the testing sites and were promoting that. We were ensuring that our community also got quarantine support, so if they needed it, they could go through the county – we had some funds from the county – or individually [deidentified]got funding through different foundations to ensure that people got economic support and that was part of our response. (CM19-CM21)*

CE/COs evolved with the pandemic response: anticipating needs, bundling response strategies, and ultimately embedding these in programming. The functional-operational-structural capacity cycle was part of a responsive, community-centered, and nimble approach to challenges faced by immigrant communities.

#### Community attitudes and perceptions

CE/CO awareness of the circulating attitudes and perceptions about COVID-19 within the community guided and facilitated the translation of community voices into action steps. CE/COs described how fear and sense of responsibility drove the actions of community members. Fear was expressed in relation to concerns about livelihood; protection and care of family; isolation resulting from the closing of places of worship and loss of cultural gathering traditions; and reflections on the disproportionate impact of COVID-19 including economic inequity and misinformation.

*Folks don't have health insurance. There was fear at the beginning of the pandemic; there were bills coming back to people who had gotten tested. In communities like ours*, [when] *something good happens, everyone in town will know what's going on. But if something bad happens, word gets out even faster, what goes wrong. That has been, even until now a barrier. It's not been accurate information. (CM19-21)*

An interviewee compared observations from previous work in lead poisoning prevention with immigrant families, to observations of current fear-driven responses of families.

*I was working within public health with some other organization…this organization was never able-able to communicate this message properly on behalf of public health…they didn't know culture, how they behaved, why they behaved. As soon as we got that contract from them I literally told them that, hey, this is a population that – these are people that literally they are my family, my friends, I go to their houses and let me do this. I don't want to do it because of the $50,000 grant but because this an urgent public health issue in our community. We strategized, we sent for our community, we brought our elder, we brought our religious scholars…and we talked with people and they're afraid. The main reason that they don't take their kids to get tested was that they thought that the Childcare Protective Services would come take your kid because you put them in that situation that they have a high level of lead. So they hid. (CM05)*

In contrast to threads of fear were narratives of humanity and resilience, where community supported community and emphasized the protection of one another. Interviewees shared observations of informal contact tracing, where individuals who tested positive would self-notify families and friends they had been in contact with. One interviewee (CM09) emphasized that it was not mistrust in the government or health system driving these actions, but rather a conscientiousness around the safety of contacts and preventing broader community exposure. An interviewee described dynamics in younger immigrant communities that promoted a sense of responsibility and giving back.

*The immigrant population of* [deidentified] *is very young, in their 20s and 30s, and a lot of the young individuals that come in, they're coming to make money to send back to their countries. They form families, they start having babies, so it's a very young, new, open generation that not only is here to work and make money to send back to their countries, but they also want to build their own American Dream, right?…The biggest thing that I've noticed with our* [deidentified] *population is that they are wanting to give back to the community. So, these 14 individuals that I'm telling you that I have as community health workers or promotores, the majority of them, we helped them some way, somehow, and they've wanted, they've been wanting to give back, so they volunteer their time to help us. (CM01)*

CE/COs reflected on how a shared value of looking out for one another guided community action during the pandemic.

*I think it's important to know that our community, they're resilient. They take care of each other. They really try to take care of each other, and when the system falls, we all help each other, all try to, you know, “Hey, this person has Covid-19, so let's all go take some food,” or something. We all try to help each other that way. We're extended families, relatives that, it doesn't matter if my grandmother and my great-grandmother, they're first cousins or something; we're a close bond, you know…we don't believe in leaving somebody alone only. In deaths or anything like that, we're always there. (CM15)*

Community context influenced CE/CO approaches during COVID-19 through an understanding of the history of the communities served, how community attitudes and perceptions evolved across the trajectory of the pandemic, and ways organizations themselves pivoted to reflect the community-expressed needs.

### Orientation

The CE/CO orientation to *process*, emphasizing intention and how activities were approached, was characterized by commitment, adaptation to community needs/attitudes/perceptions, and presence. One interviewee stated…*we are still here…we are like essential workers serving essential workers (CM16-17)*. Generational and sociopolitical orientations were important factors informing tailored and targeted processes.

#### Generational orientation

CE/COs acknowledged the strengths and challenges unique to generations, important interactions between generations in the community, and ways that CE/COs integrated this understanding into their work.

*Understand that it takes time for people sometimes to adapt to this new world, new culture, and being patient with us. Some people, they expect us to come here, within a few months, a year or so, just be on point and know everything, know where to go for things, and sometimes that's not the case, especially when you're dealing with elderly people. Young people, they're OK because they can adapt faster. They go to school; they interact with others, but elderly people or sick people don't have the ability to connect how young people connect, social media and stuff like that, and are more closed off, introverted and don't really communicate with others unless it's people from the same community. (CM08)*

Isolation and a lack of cultural connection were well-documented challenges during the pandemic among immigrant communities, where cultural traditions and rituals often centered around community gatherings. In many spaces, social media served an essential role, not only in the dissemination of information, but in re-establishing the connection. Interviewees noted that access to technology was a barrier among older members of communities. Young people in communities functioned as a bridge, helping the older generation to access information related to COVID-19 and other pandemic resources (e.g., testing, vaccination), as well as maintaining a connection with culture and community. Said another way, *There is a reliance on the younger generation to see things on social media and share with families (CM23)*. Generation-related observations informed the work of organizations to foster connection.

One interviewee described a balance between the process of doing CICT effectively and efficiency in the context of generational considerations.

*But then for the older generation, elders, it* [case investigation] *can take up to an hour because, like I said, they like to tell you their stories and how they're feeling. You just have to be willing to be on the phone with them, just to listen to them so they're opening up to you. That's how you gain their trust, too, if you're not rushing them. Like I said, you have to have that sincere tone when you're talking to them. (CM22)*

The ability to understand generational perspectives and integrate this into an approach to CICT required a deeper understanding of lived experience. Trust was fostered at the intersection of process and intentionality.

#### Sociopolitical orientation

Circumstances related to economics, politics, and gender that were inherent to the migration experience in the United States informed CE/CO approaches. CE/COs were not themselves political entities. But interviewees described the need to respond to the highly politicized nature of the pandemic response through programmatic shifts in service delivery in order to mitigate sensitivities around immigration status and documentation. Sociopolitical context was one of the areas where perspectives of public health and perspectives of CE/COs were most discordant. Concerns about how immigration status influenced access were made implicit and explicit by interviewees. These concerns were referenced or alluded to in discussions around testing access and other COVID-19 services, housing, eligibility for stimulus payments, and other forms of economic support. CE/COs were involved in and supported individuals who were weighing difficult decisions around public health guidance and sustaining their families.

*When we decided to do this testing, it was anonymous and taking all barriers from the people, that we were not going to be asking for insurance, we were not going to be asking for employers; all we were asking was, if you're interested and you want to take it, just give us a contact number, your phone number, where we would send you the results. (CM16-17)*

CE/COs were constantly adapting activities and priorities based on how identities and lived experiences were discussed, measured in data (being seen and being counted), and acknowledged as critical to health justice. For example, the importance of acknowledging multilingual community members and language justice, a framework emphasizing the complex role of language in equity.

#### Service delivery models promoting access

CE/COs adopted approaches that allowed rapid and nimble pivots in service delivery. These service delivery models, organizational partnerships, and coalition-building activities supported CE/CO ability to serve communities despite the variable availability of resources and support. At times CE/COs learned and innovated independently from public health and health systems during the pandemic, using existing knowledge to adapt efficient, culturally rooted systems of service delivery. Most notably, interviewees described robust community health worker (CHW) implementation models and the impact of those services.

…*CHWs would get those names and reach out to those individuals that tested positive. There's a section with a question that asks what language do you prefer, so all the* [deidentified]*-speaking community members are picked up by the other CBO that is working with the* [deidentified] *community. I feel like this COVID Equity Project has been really helpful, geared toward the community members. (CM22)*

CE/COs highlighted intentional approaches to CHW training and professional development, specifically, where communication, sincerity, patience, and trust building formed the foundation of the interactions with community members. CHWs identified and met generational needs related to communication platform, time allocated and spent with older members of the community, and the various ways the “need to feel heard” was manifested across age groups. CE/COs described a perceived underutilization of CHWs by the public health system—particularly in circumstances where CHWs could have quickly pivoted to meet an unaddressed need.

*I think just having the right staff trained for the communities that they will be working with is really helpful. Like I said, they know the communities, they have the language, they know how to speak to the communities to have them have that trust. And just to be able to listen to them when you speak to them, because maybe they don't get a lot of phone calls. “We just want to call and listen to you, your stories,” especially from the elders. They're lonely, so if people don't call and talk to them… They've gone through so much in life, running away from the* [deidentified] *War and the transformation from the refugee camps to the United States. They've just gone through so much, so I think just having the right team and have them trained and to have them listen to them, it would really open up more conversations with community members, so that they have trust in the organization that you're working with. And then if you're offering resources, offering other things for them, make sure to follow up. Don't just leave and say maybe they got the resources; maybe they didn't. Make sure to follow up with them, so that they continue to have the trust in you. (CM22)*

### Relationality

CE/COs pursued authentic and sustainable connection to the community and reflected on the relationships between local public health, the CE/CO, and community. Dynamics of advocacy and partnership were complex. The characteristics of these relationships either widened gaps or challenges or helped to bridge gaps. Despite good intentions, a lack of clarity and/or inconsistent actions and messaging ultimately undermined efforts and the formation of trust. Dependability was important.

…*I get calls from people that tested positive and public health has not reached out to them, and then I get calls from people, “They said they would call me back, and they have not. They took our list of what we've really needed, and we've not gotten nothing,” those kinds of things. And because I am a cultural navigator with public health, I ask them, “How can we help? Because people are calling us. How can we help?” We understand there's a pandemic and they're so overwhelmed with everything, but utilize us as people that are directly in contact with the community. But there's all kinds of bureaucratic stuff. (CM08)*

Interviewees described how consistency and authenticity fostered trusting relationships.

*We have gone to knock on doors. We knock on doors, especially where we know our community concentrates, so we have specific neighborhoods that we go and do some door knocking. We do calls, so we call the lists of contacts that we have. We also go to little stores, especially in the rural areas in the different counties in the* [deidentified]. *We go in and do a table at one of the community stores or laundromats. We go and talk to the community where we know they're still going, not to pass out information at events, but just to have those conversations as people are coming to those places. (CM19-21)*

Relationality was achieved through deep connection and knowledge of community, consistency, and presence. As CE/COs reflected on the characteristics of authentic relationships, they shared the value of trusted messengers, for example, community leaders, as information pathways.

*It's easier to believe the community leader than the CDC… So if the CDC or public health is contacting this guy or the leader about it and talk to him. There's a lot of things, culture, society, religion…But if the community leader can talk to them, it's easier to believe him than the others* [public health]. *If they believe and trust him, they'd be like, “I can go ahead and do that.” (CM26)*

CE/COs also described community leaders as trusted messengers through mutuality and partnership.

…*Going into those communities, because you have to show up, too. Not everything can be done online, so you have to be present in those communities and connecting with those leaders in those communities to be able to have that partnership with them. They are trusted community members, so they can relay the message to the larger group. What else? Trust… You have to be present. I'm sorry, but we don't see people. People don't come to us. They don't come to our communities and talk to us, share those resources. They may post it on their website or go to a community event in another area, not where my people live, so they don't know about those things. Yeah, being present, coming into our communities and connecting with those religious leaders or ethnic leaders. (CM08)*

Schools were also perceived as important trusted messengers. Interviewees acknowledged that existing relationships between schools and public health, the consistency of school messaging, and the qualities of the relationship between schools and families facilitated the transfer of information about COVID-19 within the community.

…*People are receiving dependable information. They receive a message or text message about the schools linked with the COVID-19, so that's really important and families are always participating in the schools' Zoom meetings…the school system is really good because they can reach each family because they have their system. When they* [schools] *receive any of this from the health department, they put their own information on top of that and send that to all families. They* [schools] *have interpreters and translate all those materials into the languages that people speak in the community and send it to them. So every family, if they have kids enrolled in school, they receive updated information every day. (CM09)*

Social media functioned as an essential platform for connection with and within immigrant communities. Social media was prominent in statements describing means to connect systems or institutions and community. CE/COs acknowledged the importance of social media for information, the potential for misinformation, and therefore, the need to balance this with communication approaches that promote mutual understanding.

Key components of communication for multilingual communities include language access and language justice. Interviewees reflected that even before the pandemic, language access and language justice presented significant, systemic barriers to accessing information and reinforced the invisibility of immigrant communities within systems.

*We see our communities really being left behind and not their different needs being addressed. Many of our community members don't know how to read and write. Many of them may only speak or feel more comfortable with their native language and the information around COVID has only been in the major languages, so in Spanish or English. It's always assumed to do COVID testing and even the COVID vaccine has been, register to take a test. To do that you need to have an email; you need to be able to navigate the Internet. So it's always assumed that people, one, know how to read and write, two, that they know how to use the Internet, and three, that they even have access to Internet to do it and that they know how to use it and navigate those sites. There's a lot of those assumptions and I don't think it's only true for* [deidentified] *communities, but many other immigrants or refugees that don't speak English and they don't have access. (CM19-21)*

Limited language access impacted each step necessary to get tested or vaccinated and reinforced the disproportionate impact of COVID-19 on immigrant communities. Further, when CE/COs found a lack of emphasis on language access, language justice, and trust, they observed hesitancy to disclose personal information about COVID-19 and the perpetuation of stigma about COVD-19.

*But it feels like if you don't have those relationships or if people didn't know how you were using these numbers, “why am I telling you?” There was that hesitancy to disclose who you were close to. Again, it goes back to how it's messaged and who calls. There needs to be enough awareness of why am I providing this number for you to call, and the stigma around having COVID. (CM19-21)*

Relationality in terms of community connection and trust is related closely to presence or engagement within the community.

### Presence

CE/COs explained the types of engagement or presence they pursued or observed in the pandemic response. These included: authentic presence, conditional presence, and lack of presence. CE/COs described the attributes of these presence “types” and how community members felt the presence. The presence was a key component of how CE/COs supported the process of CICT rather than seeking transactional information or service transfer. Interviewees reflected on attributes of presence, like frequency, consistency, and visibility. They expanded on the role of trusted messengers and described developing and sustaining connections through presence.

In describing authentic presence, CE/COs emphasized supporting the process and relationship building rather than information transfer. They reflected on the role of listening, an approach that facilitated mutuality in identifying and working toward solutions in and with communities. At the core of authentic presence, interviewees described that their presence in the community was because they were the community (CM16-17). CE/COs also explained observations that contrasted how they perceived or experienced authentic presence. One interviewee reflected on their participation in a government task force noting,…*They're there, but at the same time it feels like they're not (CM20)*. Authentic presence was often equated to sustained physical presence.

Interviewees communicated frustration when presence felt lacking or conditional in comparison to the engagement of CE/COs at the frontline work of the pandemic.

*Come often – the frequency. Don't just do one event and be gone. You know what I mean? Because it's the pandemic and you're just doing pandemic-related stuff and we won't see you again until 10 years from now, 20 years from now when there's another pandemic. Be present, be consistent with the community. Don't just do drop-ins. (CM08)*

Presence included visibility and accessibility. The presence of individuals who were not trusted partners, and a demonstrated lack of understanding of how particular partners may be perceived as unsafe, undermined trust.

*We were not involved at all, but they hired this ex-military group to do kind of like the security. Imagine, when you're working with an immigrant/non-documented community, how would that look to you, and the food was not culturally appropriate… I have requested whether they have evaluated the process and it's just gone into a black hole, my request. (CM23)*

This interviewee described an effort by a local jurisdiction and health system to provide housing for individuals isolated after a positive COVID-19 diagnosis. The CE/CO was not involved in the setup and was critical of decisions they felt perpetuated tension and hesitancy in the community, i.e., the ex-military group as security, specifically because of the prevalence and sociopolitical context of irregular migration in the community. The interviewee went on to say, …*They actually left. They* [the jurisdiction] *put them* [community members] *in and they left the next day. (CM23)*

Genuine connections fostered mutual partnerships through physical presence and consistency. CE/COs acknowledged circumstances where partner groups appeared motivated by image or reputation in the media or in the community. This conditional presence was detrimental to trust-building and collaboration.

*I made phone calls to the* [deidentified] *and they said, “No, we don't have the capacity to go there.” I was really disappointed, and then when they noticed, because it was in the newspaper…they called me back and they said, “We think that we are going to be able to do that…So you have to push the people, right, in order to react.” (CM25)*

The disproportionate distribution of resources for COVID-19 among BIPOC and immigrant communities emphasized the need for equity focused and culturally responsive service provision at the center of the pandemic response. CE/COs described employee safety, equipment provision to frontline workers, and access to medical care as inequitable components of the pandemic response, which later included CICT, testing, and vaccination.

*Again, people think about, when they put public health measures and all that, they don't think about our population. They think about the general, again, white-collar population with that, “Okay, you can do this, you can do that. Oh, use your hand sanitizer, your Lysol.” Sometimes you don't have money to buy food. Are you going to spend $4, $5, $10 that they were spending on Lysol? They're not. So a lot of the public health messages are not realistic, are not sensitive to the real needs of the people that are most hit. And that's what we saw. That's what we did. That's what we responded. (CM16-17)*

The inequitable distribution of resources to address the pandemic included limited funding and a lack of an advocacy platform.

*It's so frustrating because we all know what our community is needing, so we all put our money from our own pockets into a thing to make a basket to take. There's funding out there. You hear about all this funding and then, “Why can't they help our organization to help our people?” (CM15)*

### Impact

CE/COs summarized approaches to key challenges, broadly categorized as related to access, isolation, mental health, and fear. The impact of these approaches on key challenges was dependent on context and reached across individual, community, and system levels. Interviewees described the strengths-focused CE/CO solutions to the identified challenges. Solutions included ensuring multilingual language access and multilingual messaging; leveraging or empowering student groups and professionals; providing technology support to elderly and/or low literacy communities in innovative spaces and identifying artistic outlets.

CE/COs described the position of women in communities facilitating impact. Women were pivotal in families and communities as disseminators of knowledge and awareness.

…*When you educate those women within our communities, we're able to talk to our spouses, our brothers, our sisters, our daughters, our little kids and share that information with them when you're talking about health. And I'm thinking the whole spectrum, from diabetes, blood pressure, all the illnesses, so educating women specifically on health matters, it helps the whole family; it helps the whole community. (CM08)*

In addition to outreach efforts focused on women, CE/COs described targeted outreach toward youth. Youth were perceived as being highly skilled in technology and social media. Leveraging this skill set was a means to equitable distribution of resources in the community. In contrast, when skill sets were felt to be ineffectively utilized CE/COs felt a sense of lessened impact.

…*I get calls from people that tested positive and public health has not reached out to them, and then I get calls from people, “They said they would call me back, and they have not. They took our list of what we've really needed, and we've not gotten nothing,” those kinds of things. And because I am a cultural navigator with public health, I ask them, “How can we help? Because people are calling us. How can we help?” We understand there's a pandemic and they're so overwhelmed with everything, but utilize us as people that are directly in contact with the community. (CM15)*

CE/COs envisioned how their impact could have been optimized had they been successful in establishing engagement with collaborating systems.

“*Okay, so if you need to, you can use us and let us get the training for HIPAA, because we're there and we can help you.” It's not like we're just saying that; we can help you, because we're connected with our community. And so if we want to even help lessen the burden or the work… you know what I mean?…If that's all we need to do, then let us do it. Let us get the training, and let's get whatever needs to be done so we can help you, so this virus, this pandemic and the spread, it helps that, if we could do that. Those are the things that we are really trying to figure out what to do. But there's so much…bureaucratic stuff, policies. (CM15)*

Despite this, CE/COs amplified voices that were impacted, silenced, excluded, or targeted. They listened to what communities identified they needed during the pandemic. As they had always done, CE/COs continued to provide space for perspectives to be shared and for relationships and trust to develop. Cultural humility was fundamental in this work.

*I recommend to every one of you that you find your cultural mentor. My husband is…my cultural mentor as his own culture…So that's the beauty of that, when you realize your capabilities and your willingness. It's like, OK, I'm capable in that, but I'm color blind in this. You have to be honest*. (CM25)

Cultural concordance was foundational to impactful collaborations.

*And the organization hires individuals from the community to help our own community. We train them, we build their capacity, so we build capacity, among the community and in general in the population that we serve, to help themselves. We believe that's where our, in our mission, it's self-sustainability. We believe that the type of individuals and culture that we are, the ways that we are, we take pride in having our own sustaining system, no matter what that is. But we take pride in helping our own, helping ourselves. And contrary to what many believe, that we just come to this country to ask for services, it is the contrary. We come here to work, we come here to find ways to help ourselves, and we are willing to do the hardest jobs that a lot of people are not willing to endure. And that is the population I represent and how we serve them*. (CM16-17)

Table 2 shows examples of challenges presented to CE/COs at individual, community, and system levels and ways CE/COs generated impact through presence, relationality, and orientation. Embedded are NRC-RIM toolkits and promising practices that offer strategies for public health jurisdictions pursuing a comprehensive partnership with communities to optimize capacity in emergency public health response.

**Table 2:** Individual/community/system level challenges and problem-solution connection strategies with embedded NRC-RIM toolkit and promising practice starting points for public health jurisdictions focused on building capacity.

**Individual challenge & problem-solution**	
*That person called me crying. What will happen with my child?…If my child falls sick or if I myself fall sick, what will happen?..*.[I said] *It doesn't mean that you're going to die, but it doesn't also mean you can neglect it. You need to take it seriously, but don't cry. It will be fixed. Then she said, “oh, this gives me peace of mind.”…She started calling friends…“I am in quarantine. I was in touch with you. Please be careful.” (CM10-CM13)*	
Challenge-solution integration	Challenge: Fear of COVID-19 resulting from lack of or misinformation Solution: Access to trusted community leader
Operating theme	Relationality
Toolkit for public health	Communications
Promising practice	Cultural Navigators to Liaise Between Communities and Public Health
**Community challenge and problem-solution**	
*When there's a funeral a lot of people would participate. And just having them not being able to do that, it was very traumatizing. They gather at the mosque or pray. They wash the body. I remember calling family members*. [Family would ask] *What do we have to do? Where do we go to do that? How does it work with covid when they have to cover the body with plastic? It was a very difficult moment for many of us. (CM18)*	
Challenge-solution integration	Challenge: Loss of cultural connection Solution: Culturally situated information pathway
Operating theme	Presence
Toolkit for public health	Community Engagement Toolkit
Promising practice	COVID-19 Community Coordinators
**System challenge and problem-solution**	
[The doctors] *were not able to talk to the families because of the language barrier; they were not able to talk with them because of the time restraints…This is completely new to everybody. A lot of people who were admitted into the hospital…listed me as a spokesperson or the person to contact, so I was able to help them try to communicate between the hospital and the patients. (CM14)*	
Challenge-solution integration	Challenge: Healthcare system capacity Solution: Culturally responsive, linguistically concordant liaison
Operating theme	Orientation
Toolkit for public health	Community Health Workers - Toolkit
Promising practice	Community Health Workers - Case Examples

## Discussion

This community case study described the prominent characteristics of impactful community-led COVID-19 response strategies implemented in partnership with immigrant communities in the United States. CE/COs emphasized understanding context and a culturally responsive, process-oriented approach to a community-led COVID-19 response. The actions, processes, and outcomes CE/COs described were not necessarily new, meaning they had cycled before or were cycling currently in other contexts. A key difference in the context of the COVID-19 pandemic was that the health inequities targeted by actions, processes, and outcomes had not previously posed as acute or widespread a risk to human health.

CE/COs diligently and intentionally reimagined and recontextualized the public health response to COVID-19 in immigrant communities, frequently in the setting of scarce resources, as the pandemic circumstances evolved. CE/COs demonstrated sustained presence and support in communities, while educating, advocating, and promoting access to public health services, testing, and vaccination in culturally responsive ways. An orientation to the process was foundational to the ways that community organizations approached their work within the context of the COVID-19 pandemic, in contrast to the outcomes-oriented focus of public health and health systems. This orientation was evident in how generational differences and family dynamics were integrated into testing and CICT and how COVID-19 services were tailored around employment. Identifying and building on community strengths was prioritized over compliance. Establishing a deep sense of community context facilitated communication and the ability to rapidly adjust to meet emerging needs.

Results were aligned with the expansive evidence supporting the role of community-based organizations in sustaining community health and wellbeing. While actions, processes, and outcomes associated with the community-led COVID-19 pandemic response were predominantly in circulation at the onset of the pandemic, important innovation happened within those cycles. These innovations offered new insights into the translation of advocacy into health outcomes. For example, an organization supporting a diverse network of Indigenous farmworkers described the operationalization of the language justice framework, as a practice [(16), S. M. Ortega, personal communication, March 1, 2022]. Through language-inclusive resources and services and rights-oriented outreach, the organization promoted the sustainability of an essential workforce through capacity building, representation, and resilience. CE/COs also recognized the critical role of culturally and linguistically concordant staff in order to build trust and prevent erosion of trust, and deliver services in an equitable manner, advocating for the integration of cultural mentorship into reflective and authentic work with diverse immigrant communities [(17), J. Altamirano-Crosby, personal communication, March 1, 2022]. Broadly, CHW service delivery emphasizes health promotion and community wellbeing and is accessible, particularly where issues of equitable access and complex system navigation are problems. CHW models support capacity building in health promotion among priority communities (18–21). CHWs were leveraged extensively by CE/COs to create and implement culturally responsive COVID-19 programming in the communities where they lived and worked.

CE/COs evolved foundational practices in community-public health partnership building. Health communication cycles are dependent on the presence of credible sources or “trusted messengers” (22, 23). CE/COs described the roles of religious leaders and schools in communication and messaging as more active and bidirectional. Faith and education institutions were serving mediating roles in communication. Not only were the institutions facilitating the transmission of credible information but they were also influencing the underlying relationship between the senders and receivers of information (24). Additionally, these roles represented an important counterbalance to the prevalence of misinformation on social media (25). It is essential that public health fosters relationships with these channels in addition to wider dissemination strategies, in the interest of accurate and efficient communication dissemination. Community partnership is a well-established standard in public health preparedness. CE/COs expanded on this through descriptions of the impact of sustained physical presence in the community. For example, CE/COs promoted representation as they were physically and visibly present at COVID-19 testing and vaccination sites, sometimes getting tested or vaccinated. They described fielding phone calls and questions from community members, emphasizing the role of accessibility in presence. These approaches complemented the position of public health in relationship building and partnership.

Limitations included the role of selection bias in determining our final sample. Though our sampling frame was broad, the reach of our networks (including members of the project Community Leadership Board) may have been a limiting factor in the project team's ability to engage key groups. Engagement was more limited in HHS Regions 5–8. Interviews were conducted via the Zoom platform, which could have potentially been prohibitive for certain individuals/communities depending on broadband access, and were conducted solely in the English language. Interviews were conducted at one point in time. While this design served the objectives of this project phase, it is likely that key iterations and evolutions of the roles of immigrant-serving community structures in developing and implementing culturally sustaining programming in the context of a pandemic response were not elucidated. While we attempted to gain the perspective of multiple forms of community engagement, it was not possible to capture all. Limitations were mitigated by facets of the method and procedures that established trustworthiness and authenticity (26, 27), and the informants' fundamentally important positions, perspectives, and lived experiences.

## Conclusion

…*It's kind of sad that because of the pandemic we're finally getting noticed, you know what I mean? Like we're getting the help that the community needs, and it's sad that it took a pandemic to finally hear our words, hear our needs. (CM15)*

Interviewees defined equity as the co-creation of comprehensive and holistic pandemic response services rooted in language and culture. CE/COs explained that an equitable system honors and acknowledges the unique circumstances under which decisions to migrate were made as well as the ongoing impacts of those decisions on health. The breadth of the work and observations described in the interviews are captured in the excerpt that opens the paper, *How can you advocate for something that is nonexistent? (CM16-17)*. Through a critical examination of systemic barriers and the elevation of partnerships between communities and systems that support the visibility of immigrant communities, we broaden the capacity of pandemic response. Findings inform the next steps in the application of best and promising practices that address health inequities among refugee, immigrant, and migrant communities in the United States.

## Data availability statement

The raw data supporting the conclusions of this article will be made available by the authors, without undue reservation.

## Ethics statement

The initiative was determined not human subjects research by the University of Minnesota and exempt by the University of Washington Institutional Review Boards. Written informed consent for participation was not required for this study in accordance with the national legislation and the institutional requirements.

## Author contributions

SH and YG led analysis and manuscript development. JA-C and SO provided consultation on the interpretation of results and manuscript development. KiY contributed to manuscript sections. SA, DA, WF, SK, EM, and CT contributed to analytic direction and manuscript revision. KaY and ED-H contributed to conception and design of the study. All authors read and approved the submitted version.

## Funding

This work was performed under the National Resource Center for Refugees, Immigrants, and Migrants (NRC-RIM) which was funded by the US Centers for Disease Control and Prevention and the International Organization for Migration (award number CK000495-03-00/ES1874).

## Conflict of interest

Author JA-C was employed by WAGRO Foundation. The remaining authors declare that the research was conducted in the absence of any commercial or financial relationships that could be construed as a potential conflict of interest.

## Publisher's note

All claims expressed in this article are solely those of the authors and do not necessarily represent those of their affiliated organizations, or those of the publisher, the editors and the reviewers. Any product that may be evaluated in this article, or claim that may be made by its manufacturer, is not guaranteed or endorsed by the publisher.
